# Legitimate vs. illegitimate restrictions – a motivational and physiological approach investigating reactance processes

**DOI:** 10.3389/fpsyg.2015.00632

**Published:** 2015-05-18

**Authors:** Sandra Sittenthaler, Christina Steindl, Eva Jonas

**Affiliations:** ^1^Division of Economic and Organizational Psychology, Department of Psychology, University of Salzburg, Salzburg, Austria; ^2^Division of Social Psychology, Department of Psychology, University of Salzburg, Salzburg, Austria

**Keywords:** reactance, restrictions, threat, anger, motivation, social interaction, physiological arousal

## Abstract

Threats to our freedom are part of our daily social interactions. They are accompanied by an aversive state of motivational arousal, called reactance, which leads people to strive to reestablish their threatened freedom. This is especially the case if the threat seems to be illegitimate in nature. However, reactance theory suggests that reactance should also be aroused when people are exposed to legitimate freedom threats. In this article we first aim to show that both illegitimate and legitimate freedom threats evoke reactance. Second, we aim to extend past work on reactance by exploring the underlying process of experiencing a legitimate vs. an illegitimate restriction. In the current study (*N*= 57) participants were restricted in an illegitimate (unexpected and inappropriate) or legitimate (unexpected but appropriate) way, or were not restricted at all. We assessed participants’ experience of reactance, their behavioral intentions to restore their freedom, their approach motivational states, as well as their physiological arousal (heart rate). Results indicated that when restricted in an illegitimate or a legitimate way, participants indicated the same amount of reactance as well as anger. However, when looking at people’s physiological reactions, important differences between illegitimate and legitimate restrictions become apparent. Illegitimate restrictions led to an immediate arousal, whereas legitimate restrictions led to a time delayed arousal. This suggests that illegitimate restrictions lead to a sudden increase in aversive arousal. Legitimate restrictions, however, seem to be associated with a more cognitive process in which people first need to structure their thoughts and reflect upon the situation before getting into the feeling of reactance in a physiologically arousing sense. Moreover a mediation analysis could show that behavioral intentions to regain one’s freedom result in positive and negative approach motivation. In sum we propose a combined dual-process and intertwined-process model explaining people’s reactions to legitimate vs. illegitimate restrictions.

## Introduction

Imagine the following scenario: you are a future University student and very excited about beginning your studies next month. Browsing through the rental offers in the internet, you come across an advertisement of a lovely flat right next to the University. You are totally excited about the flat meeting your expectations and you call the number given in the ad. The landlord answers your questions, gives you some information on the flat and the rental contract, and finally invites you to a viewing. When you mention that you are a University student, the landlord interrupts you and says: “No, I won’t rent my flat to a student!” and hangs up.

What’s going through your mind thinking of the described scenario? Do you feel restricted in your freedom? Do you suddenly become angry? Do you maybe recognize a slight tension in your body? Why do you feel this way? Would you feel differently if the landlord had explained to you that his past tenants had been students who had trashed and damaged the flat before leaving? Would you still be angry? Would an additional justification like this override the clear restriction you faced as a student trying to secure accommodation?

In the current study we investigate how freedom threats in social interactions shape people’s subsequent reactions. Furthermore, we are interested in investigating how people react when being restricted in an illegitimate vs. legitimate way. By analyzing people’s experience of threat, their behavioral intentions, their self-reported affect and motivation, as well as their physiological arousal, we aim to explore how reactance processes in social interactions emerge. Thereby we aim to complement Dillard and Shen’s (2005) description of reactance as an intertwined affective and cognitive phenomenon by adding the important factors of motivation and physiological arousal to increase our understanding of reactance processes.

### Reactance Theory

In his publications on psychological reactance, Brehm contends that restrictions lead to reactance, a so called “motivational arousal” to re-establish the threatened freedom (Brehm, 1966, 1972; Brehm and Self, 1989). The desire of being ‘free’ again seems familiar to almost all of us. It is about daily moments (e.g., dress regulations) of perceived and actual freedom restrictions that end up making us feel unwell, nervous, and angry. According to the degree of the perceived restriction, to the context of the restriction, and to the meaning of the restriction for our future, we basically react differently (Brehm, 1966; Brehm and Brehm, 1981).

Restrictions often arise in a social interaction context, for example in our introduction example the restriction happens in the interaction between the landlord and the student. Restrictions to people’s freedoms in social interactions have shown to trigger different reactance effects, like counter arguing, source derogations, aggression, or changes in attractiveness (e.g., Brehm, 1966; Clee and Wicklund, 1980; Brehm and Brehm, 1981; Fitzsimons, 2000). Dillard and Shen (2005) have summarized these reactions in their intertwined-process model, proposing that reactance is an intertwined process of negative cognitions (i.e., expression of disagreement with the restriction), and anger affect (i.e., feeling irritated, angry, annoyed, and aggravated). Testing competing conceptualizations of reactance, Rains (2013) confirmed the proposed intertwined-process model in a meta-analysis of 20 studies. Compared to single process models, a linear affective-cognitive model, and a dual-process model, the intertwined-process model best fitted the data.

However, what exactly is reactance if it is an intertwined combination of anger affect and cognition? If we think of dual-process models and their distinction between a more impulsive, affect driven, and a more cognitive dominated reflective modus of social behavior (e.g., Strack and Deutsch, 2004, for overview Gawronski and Creighton, 2013) – is this distinction meaningless in the context of reactance? Or can we distinguish between more impulsive vs. more reflective reactance processes? Furthermore, as reactance is defined as a motivational state (Brehm, 1966) we also need to better understand the role of motivational variables in addition to cognition and affect within the reactance process. Physiological arousal can be seen as a factor connected to motivational processes (e.g., Zanna and Cooper, 1974; Baum et al., 1986). Therefore, in the current research we compare physiological arousal following different kinds of freedom restrictions (illegitimate vs. legitimate restrictions) which should, according to Brehm (1966), both induce reactance.

Brehm (1966) suggested, for reactance behavior to emerge, the perceived restrictions do not solely have to stem from illegal acts. Restrictions that have legitimate justifications or even lead to a positive outcome can trigger reactance as well. This means that a loss of freedom, no matter how well justified should still arouse reactance. However, while illegitimate behaviors are unexpected, inappropriate, improper, and unjust, legitimate behaviors are unexpected but appropriate, proper, and just (Tyler, 2006; Zhang and Sapp, 2013). In this connection, fairness research suggests that decisions by authorities who are perceived of being legitimate (i.e., appearing fairer and appropriate) are more likely to be accepted by the people affected from the decision (Tyler and Huo, 2002).

To come back to our landlord example, one might ask whether the student may experience the same amount of reactance or less reactance depending on whether the landlord gives a justification of his behavior (legitimate restriction) or does not give such a justification (illegitimate restriction). In addition, when looking at physiological and motivational measures, there might be different kinds of processes when people are confronted with different restrictions to their freedom.

### Physiological Arousal and Motivation

Cardiovascular responses such as heart rate (HR) are believed to be the best indicator of effort effects (see Wright, 2008) and in general to be a particularly promising method for showing physiological arousal in human beings (Brehm et al., 1964; Croyle and Cooper, 1983; Elkin and Leippe, 1986; Losch and Cacioppo, 1990; Harmon-Jones et al., 1996; Robinson and Demaree, 2007; Butler et al., 2009). Additionally, research showed that effort influence on the cardiovascular system is mediated by sympathetic nervous system activity, which is especially well reflected inter alia in heart contractility changes (Papillo and Shapiro, 1990; Berntson et al., 1993; Brownley et al., 2000). Similarly, Baum et al. (1986) already showed that reactance is associated with an increased activity of the sympathetic nervous system. Other studies related to learned helplessness demonstrated that the factor decisive for the increased physiological arousal, which is associated with one’s motivational intensity to take action, is one’s expected coping potential (Wortman and Brehm, 1975; Brehm and Self, 1989; Brehm, 1999). In studies testing Wortman and Brehm’s model on reactance and helplessness (Pittman and Pittman, 1979; Mikulincer, 1988), a threat led to depression if people did not expect to be able to take action but aroused anger if people expected to be able to take action.

Consistent with these findings, research has shown that the possibility for coping with a situation is also related to approach motivation (Harmon-Jones et al., 2003). Approach motivation is a state in which people are motivated to move toward something and as such is related to not only positive but also negative emotions (Harmon-Jones et al., 2013). In several studies, Harmon-Jones et al. (2009) demonstrated that anger, a negative affective state, is related to approach motivation (e.g., Harmon-Jones and Allen, 1998; Harmon-Jones et al., 2003; Harmon-Jones, 2004). However, this was found only for conditions in which people were given the opportunity to resolve the anger-arousing event (Harmon-Jones et al., 2003, 2006). If reactance is aroused one believes that he or she could engage in behaviors to restore freedom and thus, approach motivation should emerge. Additionally, the emotional state of anger is a central component of reactance (Dillard and Shen, 2005; Rains, 2013) and thus, reactance should lead to a motivational state of approach. This approach motivational state should then result in people’s reactant behavior, which means that they try to approach the restoration of their freedom.

### The Present Research

In the present study we aim to investigate differences in illegitimate and legitimate restrictions by showing that different processes are activated in people experiencing those different restrictions. In line with Brehm (1966), we predict that both, individuals who experience illegitimate as well as individuals who experience legitimate threats should experience more reactance than a control group in which no threat occurs (hypothesis 1). Given Dillard and Shen’s (2005) intertwined-process model in which anger plays a central role in all reactance processes, and the literature on anger being associated with approach motivation (e.g., Harmon-Jones et al., 2003), we further hypothesize that both restrictions evoke a negative approach motivational state, namely anger (hypothesis 2). So far, we assume that illegitimate and legitimate freedom threats are approximately similar concerning their outcomes. However, is there any difference regarding the physiological process underlying people’s responses to freedom threats? Building on the dual-process model by Strack and Deutsch (2004), we hypothesize that illegitimate and legitimate threats differ in the way physiological reactions are aroused: as an illegitimate restriction does not give any justification for the threat, people should immediately feel the urge to restore their freedom. This should be reflected in an increase in physiological arousal immediately after experiencing the restriction. Therefore, an illegitimate restriction may trigger a fast process immediately leading to the motivational state of reactance accompanied by strong approach motivation. In contrast, as legitimate restrictions usually give justifications for the threat, they first may not be experienced as arousing as illegitimate restrictions. People might first need some time to reflect upon the restriction and its justification and to find counter-arguments (Dillard and Shen, 2005). We thus predict that legitimate restrictions are accompanied by a more cognitive process in which people first think about the restriction leading not to a similar immediate increase in physiological arousal as predicted for illegitimate restrictions. However, as both legitimate and illegitimate restrictions are predicted to lead to reactance (Brehm, 1966; Miron and Brehm, 2006) we propose that legitimate restrictions are also arousing but only after a delay of thinking and counter-arguing (Dillard and Shen, 2005). We hypothesize that while illegitimate restrictions trigger a more emotional, faster process and thus, an immediate physiological arousal, legitimate restrictions trigger a more cognitive, slower process and thus, a delayed physiological arousal (hypothesis 3). However, both restrictions should result in increased anger (and negative thoughts) as predicted by the intertwined model by Dillard and Shen (2005). Anger can be seen as an indicator of approach motivation (Harmon-Jones et al., 2013). As any attempt to solve a threat is an attempt to restore one’s agency and thus, one’s approach motivation (Jonas et al., 2014), we should find an approach motivational state at the end of the reactance process. As approach motivation is independent of valence we predict that behavioral intentions to regain one’s freedom should result in the experience of a positive and negative approach motivated state (hypothesis 4).

## Materials and Methods

### Participants and Design

In this laboratory study, 57 students (41 female and 16 male) with a mean age of 22.51 years (SD = 4.94, two missing data points) of the University of Salzburg, Austria, voluntarily participated. The students had been recruited in several psychological lectures and at the University campus where they were asked to participate in a physiological laboratory study. Students were randomly assigned to one of the three experimental conditions (illegitimate vs. legitimate vs. control).

### Experimental Procedures

Participants in the experiment were asked to participate in a paper-and-pencil study for approximately 25 min, while we recorded physiological measurements. The experimenter explained that she was interested in what happens physiologically when students read about typical situations in students’ daily life. The students were asked to complete all questions honestly and silently and were informed about the voluntary nature of participation as well as confidential use of data.

The questionnaire started with some demographic questions about sex, age, and field of study. After filling out these questions, the experimenter attached three “sensors” to the participants’ fingers on the non-dominant hand to measure skin conductance (SC) and HR during the study. The fingers of the participants were washed with alcohol before attaching NeXus 10 sensors (two measuring SC fixed with Velcro^®^strip and one single finger clip measuring HR) on the first, second and third fingers. Participants were told to try holding their hand as still as possible while filling out the questionnaire. First a 3-min baseline measure ensured participants would fully focus on the study. They were told to calm down and try to relax for the next 3 min looking at the black screen of the computer. Then participants were asked to carefully read and imagine the reactance-arousing scenario (3-min period). In the illegitimate condition, they were asked to imagine that they were going to start studying at the Paris-Lodron-University in Salzburg the following semester, and were therefore looking for an apartment near the University. In a press advertisement they found an appropriate 1-room-apartment downtown. They called the landlord about viewing the apartment. When the landlord asked them for their profession, they stated they would be a student in Salzburg next semester. Before they could say anything else the landlord interrupted them and stated: “No, you are a student, you won’t get this apartment” and broke off the call *(illegitimate condition)*. By contrast, in the *legitimate condition* the volunteers were asked to think of the same situation described above, but after the landlord had interrupted them he explained his behavior why he did not like students to rent his apartment. He explained that he was very sorry for his behavior but that he had bad experiences with student tenants in the past. In the *control condition* the students were asked to imagine that s/he was able to rent the apartment without experiencing any restrictions.

After participants had read the apartment search scenario, we assessed participants’ state reactance. The items were arranged into two different scales: experience of reactance (α = 0.89, seven items, e.g., “To what extent do you perceive the reaction of the landlord as a restriction of freedom?” and “How much pressure do you feel as a result of his reaction?,” adapted from Jonas et al., 2009), and behavioral intentions including evaluation items (α = 0.84, 10 items, e.g., “To what extent would you describe this man as incompetent to other students?”; “Would you like to ruin this landlord’s reputation by publishing a negative review on a respected internet site?”). Answers were given on a 5-point Likert-type scale from 1 (*not at all*) to 5 (*very much*)^1^.

For the dependent measure of the physiological arousal in this study we used SC^2^ (in micro-mho) and HR (in beats per minute). Concerning future calculations with the physiological arousal we differentiated an immediate response to the threat of freedom while reading the scenario and a delayed response while answering the reactance items, which followed the apartment-search scenario. We used the difference values between both the immediate response and the baseline measure (ir-bm), and the delayed response and the (dr-bm) for further calculations. These difference values served as our measures of physiological arousal.

We assessed people’s approach motivation by using the PANAS (Positive and Negative Affect Schedule consisting of a negative and a positive emotions scale; Watson et al., 1988). However, based on prior research showing that some of the PANAS items are related to approach motivation (Harmon-Jones et al., 2009; Steindl et al. in preparation) we created a measure for negative approach motivation, namely anger (upset, hostile, irritable; α = 0.85), and a measure for positive approach (active, attentive, inspired, alert, interested, strong, determined; α = 0.73)^3^.

At the end of the study, participants were debriefed and thanked for their participation, and received course credits if desired^4^.

## Results

### Reactance Measures

**FIGURE 1:**
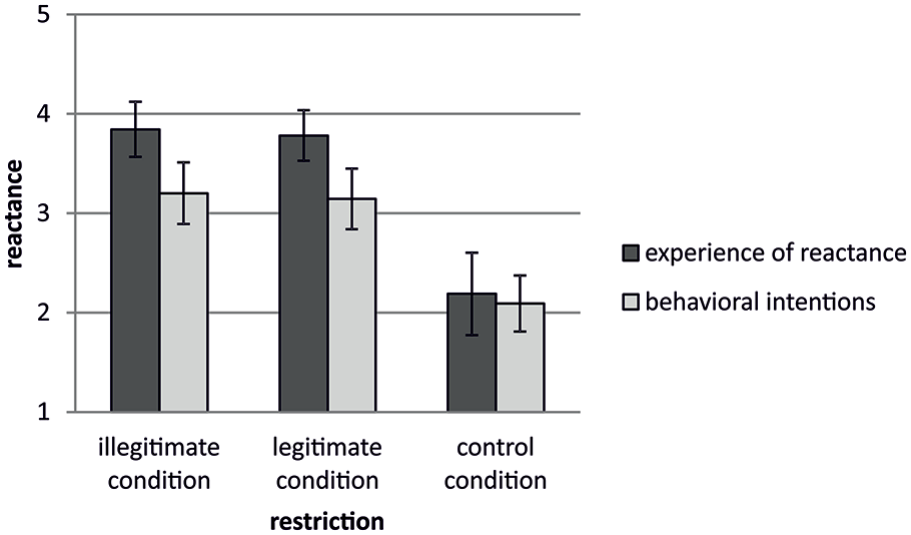
**Experience of reactance and behavioral intentions in the three experimental groups**.

To test hypothesis 1 that both illegitimate and legitimate restrictions lead to reactance we conducted univariate analyses of variance for the experience of reactance and the behavioral intentions measures separately. Means and error bars (95% CI) are displayed in **Figure 1**.

#### Experience of Reactance

As expected, participants in the illegitimate (*M* = 3.84, SD = 0.58) and the legitimate condition (*M* = 3.78, SD = 0.50) scored higher on the experience of reactance measure than participants in the control condition (*M* = 2.19, SD = 0.84), *F*(2,54) = 38.94, *p* < 0.001, $\eta_{\rho }^{2}$= 0.59. Subsequent *post hoc* analyses showed significant differences between both the illegitimate and the legitimate condition compared to the control condition (*p*_s_ < 0.001). There was no significant difference between the illegitimate and the legitimate condition (*p* = 0.761).

#### Behavioral Intentions

As expected, participants in the illegitimate (*M* = 3.20, SD = 0.64) and the legitimate condition (*M* = 3.14, SD = 0.60) scored higher on the behavioral intentions measure than participants in the control condition (*M* = 2.09, SD = 0.57), *F*(2,54) = 20.38, *p* < 0.001, $\eta_{\rho }^{2}$= 0.43. Subsequent *post hoc* analyses showed that both the illegitimate and the legitimate conditions differed significantly from the control condition (*p_s_* < 0.001). There was no significant difference between the illegitimate and the legitimate condition (*p* = 0.779).

### Approach Motivation – Anger and Positive Approach

To investigate hypothesis 2, stating that both, illegitimate and legitimate restrictions evoke approach motivation, we conducted a multivariate analysis of variance (MANOVA) with the PANAS scales’ negative approach motivation (anger) and positive approach factor. We found a significant effect for anger, *F*(2,54) = 9.56, *p* < 0.001, $\eta_{\rho }^{2}$= 0.26. Participants in the illegitimate (*M* = 3.35, SD = 0.96) and legitimate condition (*M* = 3.19, SD = 1.10) scored higher on anger than participants in the control condition (*M* = 2.00, SD = 1.07), *p* < 0.001, and *p* = 0.001, but participants in the illegitimate and legitimate condition did not differ from each other, *p* = 0.628. However, we did not find a significant effect for people’s positive approach, *F*(2,54) = 2.32, *p* = 0.108, $\eta_{\rho }^{2}$= 0.08, indicating that participants in the illegitimate (*M* = 2.98, SD = 0.46), legitimate (*M* = 2.76, SD = 0.76), and control condition (*M* = 3.23, SD = 0.59) showed about the same amount of positive approach.

### Physiological Measures

#### Heart Rate

To test hypothesis 3, whether illegitimate restrictions lead to an immediate physiological response whereas legitimate restrictions lead only to a delayed physiological response, we performed a mixed-model ANOVA for the time of measurement (immediate physiological response vs. the delayed physiological response) as within-subject factor and the kind of restriction (illegitimate vs. legitimate vs. control) as between-subject factor. We found a significant main-effect of the restriction manipulation, *F*(2,54) = 3.40, *p* = 0.041, $\eta_{\rho }^{2}$= 0.11. *Post hoc* analyses indicated that participants in the illegitimate condition (*M* = 3.72, SD = 0.76) showed a greater physiological response compared to participants in the control condition (*M* = 0.90, SD = 0.78), *p* = 0.012 but not compared to participants in the legitimate condition (*M* = 2.56, SD = 0.80), *p* = 0.296. Participants in the legitimate and control condition did not differ either, *p* = 0.142. We further found a main-effect for the within factor (immediate vs. delayed response), *F*(1,54) = 13.37, *p* = 0.001, $\eta_{\rho }^{2}$= 0.20. Participants showed more physiological arousal at the second point of measurement (delayed response, *M* = 3.02, SD = 4.21) compared to the first point of measurement (immediate response, *M* = 1.81, SD = 3.29).

**FIGURE 2:**
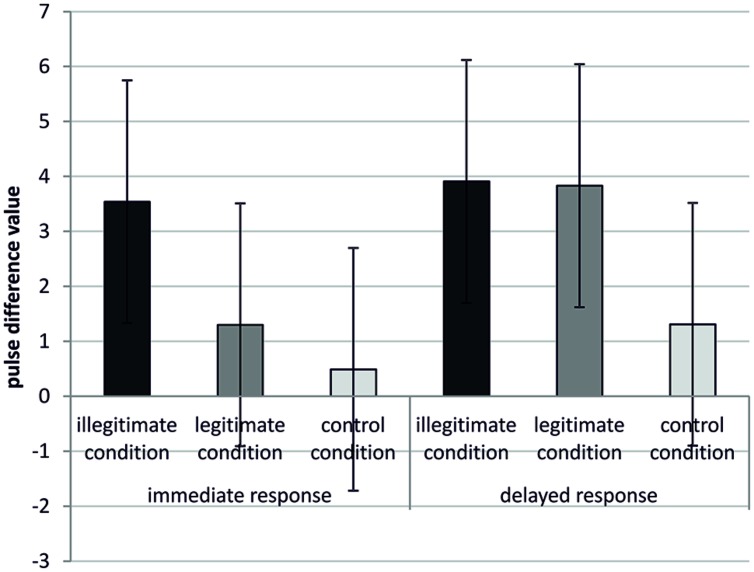
**Physiological arousal for the immediate and delayed response in the three experimental groups**.

Furthermore and most importantly, we found the predicted interaction (point of measure × restriction), *F*(2,54) = 3.67, *p* = 0.032, $\eta_{\rho }^{2}$= 0.12. Simple effects within the illegitimate [*F*(1,54) = 0.43, *p* = 0.517, $\eta_{\rho }^{2}$= 0.01] and control condition [*F*(1,54) = 1.96, *p* = 0.168, $\eta_{\rho }^{2}$= 0.04) showed that participants’ physiological arousal did not differ in the immediate and delayed response. However, within the legitimate condition [*F*(1,54)= 17.55, *p* < 0.001, $\eta_{\rho }^{2}$= 0.25], participants showed much more physiological arousal in the delayed compared to the immediate response condition. Moreover, within the immediate response condition, legitimately restricted participants showed about the same level of physiological arousal as participants in the control condition, *p* = 0.427. Participants in the illegitimate conditions displayed higher physiological arousal values compared with participants in the control (*p* = 0.003) and the legitimate condition (*p* = 0.029). Following the delayed response condition, participants in the illegitimate (*p* = 0.068) and legitimate condition (*p* = 0.053) tended to display higher arousal values compared with participants in the control condition. Participants in the illegitimate condition displayed about the same level of physiological compared to participants in the legitimate condition, *p* = 0.950. These results support our hypothesis. Means and SD as well as error bars (95% CI) are displayed in **Table 1** and illustrated in **Figure 2**. Moreover, correlations with all measured variables are displayed in **Table 2**.

**Table 1:** Means and SD for the ANOVA as a function of the experimental manipulation: immediate response and delayed response concerning heart rate (HR).

	Immediate response^a^		Delayed response^b^	
Manipulation	*M*	SD	*M*	SD
Illegitimate condition	3.54 (*n* = 20)	3.26	3.91 (*n* = 20)	4.48
Legitimate condition	1.30 (*n* = 18)	2.30	3.83 (*n* = 18)	3.17
Control condition	0.49 (*n* = 19)	3.49	1.31 (*n* = 19)	4.46

*^a^Immediate response = HR reading the scenario – baseline measure (difference value); ^b^Delayed response = HR answering the reactance items – baseline measure (difference value).*

**Table 2:** Mean and SD and correlation for all dependent variables in the different groups.

		*M*	SD	1	2	3	4	5	6	7	8
(1) Experience of reactance	Illegitimate Legitimate Control	3.84 3.78 2.19	0.58 0.50 0.84	–							
(2) Behavioral intentions	Illegitimate Legitimate Control	3.20 3.14 2.09	0.64 0.60 0.57	0.57^∗∗^ 0.60^∗∗^ 0.75^∗∗^	–						
(3) HR immediate	Illegitimate	3.54	3.26	0.07	–0.23	–					
	Legitimate	1.30	2.30	–0.05	–0.05	–					
	Control	0.49	3.49	0.18	0.02	–					
(4) HR delayed	Illegitimate	3.91	4.48	0.32	–0.02	0.82^∗∗^	–				
	Legitimate	3.83	3.17	–0.04	–0.08	0.66^∗∗^	–				
	Control	1.31	4.46	0.18	–0.11	0.80^∗∗^	–				
(5) Anger	Illegitimate	2.98	0.64	0.67^∗^	0.69^∗^	–0.20	0.05	–			
	Legitimate	2.76	0.76	0.66^∗^	0.68^∗^	–0.10	–0.21	–			
	Control	3.23	0.59	0.87^∗^	0.84^∗^	0.08	0.02	–			
(6) Positive approach	Illegitimate	3.35	0.96	–0.17	0.15	–0.17	–0.19	0.10	–		
	Legitimate	3.19	1.10	0.53^∗^	0.66^∗^	–0.39	–0.33	0.59^∗^	–		
	Control	2.00	1.07	–0.23	–0.29	–0.58	–0.44	–0.14	–		
(7) Skin Conductance (SC) immediate	Illegitimate	0.92	0.81	0.04	–0.07	0.15	–0.18	–0.20	0.28	–	


	Legitimate	0.69	0.74	–0.29	0.05	0.32	0.29	–0.09	–0.02	–	
	Control	0.77	1.14	–0.03	–0.06	0.60^∗^	0.51^∗^	–0.10	–0.48	–	
(8) Skin Conductance delayed	Illegitimate	0.55	0.63	–0.14	–0.11	0.15	–0.26	–0.33	0.24	0.91^∗^	–
	Legitimate	0.24	0.36	0.06	0.17	0.16	0.16	–0.18	0.14	0.50^∗^	–
	Control	0.24	0.53	–0.16	–0.12	0.45	0.26	–0.09	–0.38	0.86^∗^	–

*^a^Immediate response = HR reading the scenario – baseline measure (Difference value); ^b^Delayed response = HR answering the reactance items – baseline measure (Difference value); ^∗^*p* < 0.05, two-tailed. ^∗∗^*p* < 0.01, two-tailed.*

### Approach Motivation

**FIGURE 3:**
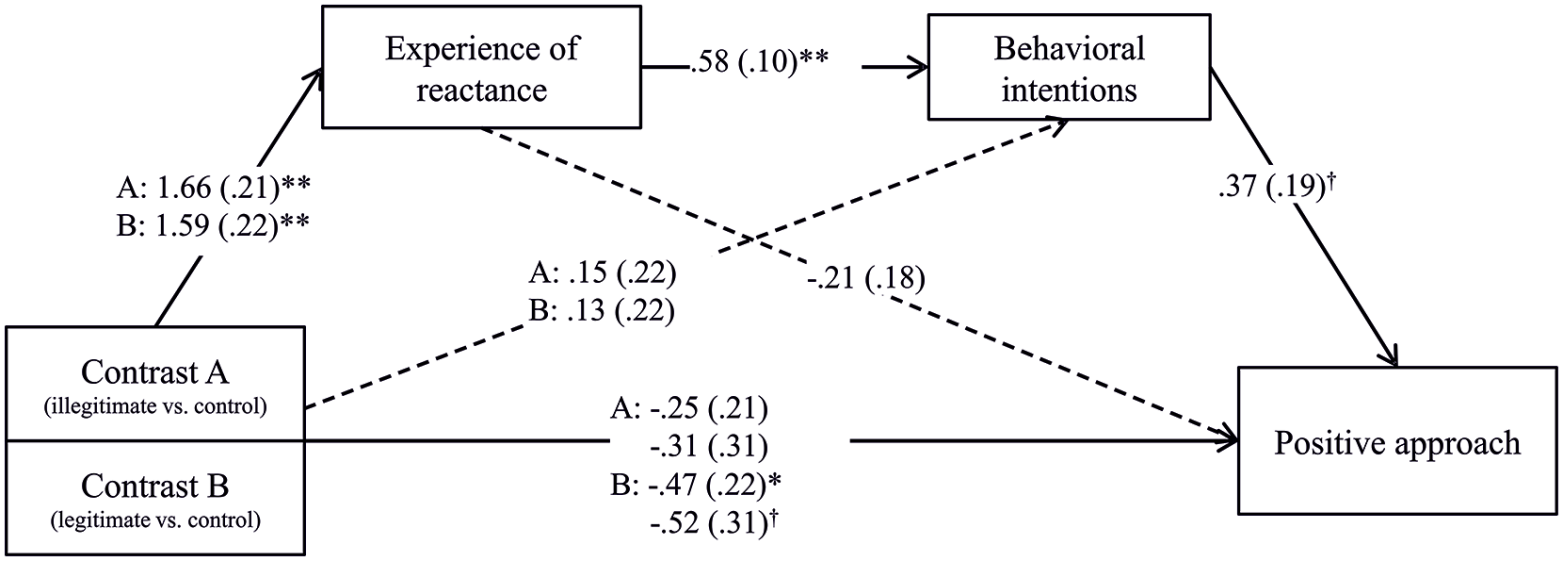
**The effect of freedom restrictions on negative approach (anger) via experience of reactance and behavioral intentions.** ^†^*p* < 0.10, ^∗^*p* < 0.05, ^∗∗^*p* < 0.01.

Hypothesis 4 predicted that the attempt to restore freedom leads people to come back into an approach state. Using the software Process 2.11 (Hayes, 2013, model 6), we performed two serial multiple mediation analyses, one with positive approach and one with negative approach (anger) as a dependent variable. First, we employed Contrast A (illegitimate vs. control condition, legitimate condition as a covariate) and Contrast B (legitimate condition vs. the control condition, illegitimate condition as a covariate) as the independent variables, positive approach as the dependent variable, and reactance experience and behavioral intentions as the two mediators. We used a 95% bias corrected bootstrap confidence interval (95% BC CI) and 5000 bootstrap samples. We found that the illegitimate threat did not have a significant total effect on positive approach, *b* = -0.25, SE = 0.21, *t*(54) = -1.16, *p* = 0.250. The legitimate condition, however, had a negative effect on positive approach, *b* = -0.47, SE = 0.22, *t*(54) = -2.15, *p* = 0.036, indicating that the legitimate restriction led to a lower positive approach. The effects for both restrictions was non-significant when the potential mediators experience of reactance and behavioral intentions had been added to the prediction, *b* = -0.31, SE = 0.31, *t*(54) = -1.01, *p* = 0.320 and *b* = -0.52, SE = 0.31, *t*(54) = -1.72, *p* = 0.091. The bootstrapped indirect effects of the illegitimate and the legitimate freedom threat via experience of reactance and behavioral intentions was significant in a positive direction, *b* = 0.36, SE = .19, BC CI [0.03, 0.80] and *b* = 0.34, SE = .19, BC CI [0.04, 0.82] (for the path coefficients see **Figure 3**). Thus, both restrictions which first arouse an experience of reactance that further led to behavioral intentions to restore one’s freedom resulted in a final positive approach state suggesting that approach had been restored.

**FIGURE 4:**
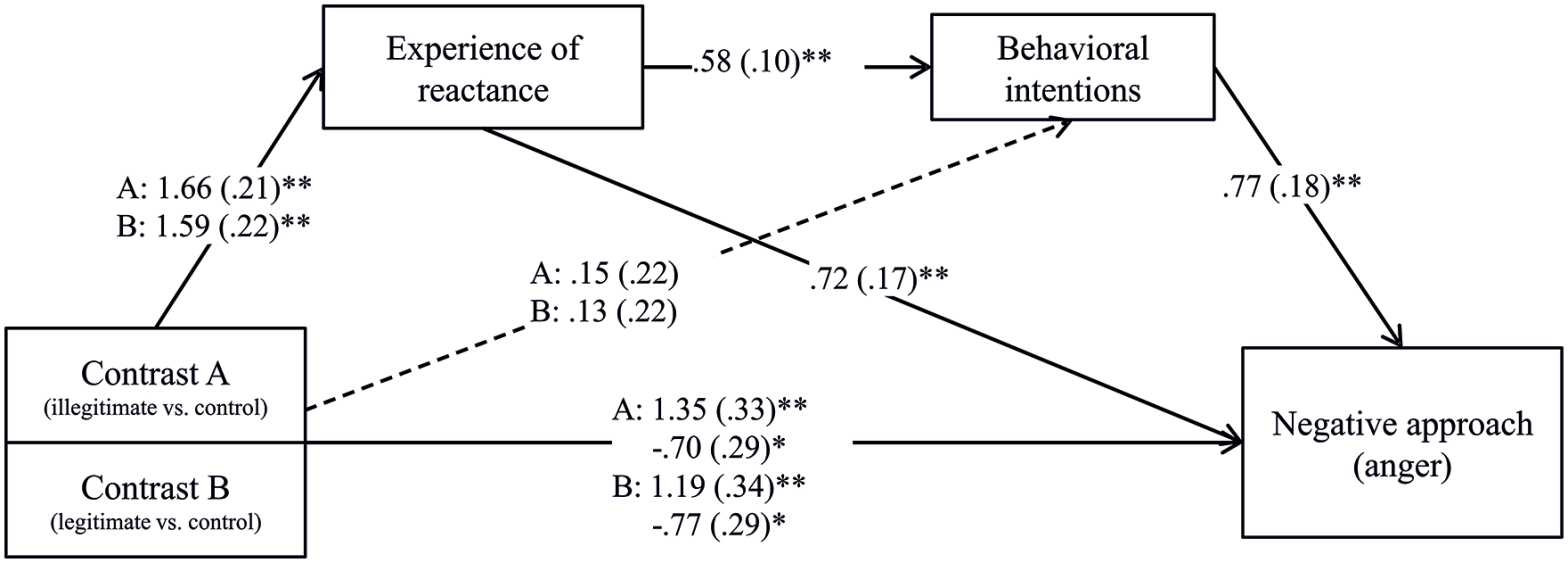
**The effect of freedom restrictions on positive approach via experience of reactance and behavioral intentions.** ^∗^*p* < 0.05, ^∗∗^*p* < 0.01.

Second, we performed the same analyses but this time with negative approach (anger) as the dependent variable. We found that both, the illegitimate as well as the legitimate threat had a total effect on anger, *b* = 1.35, SE = 0.33, *t*(54) = 4.05, *p* < 0.001 and *b* = 1.19, SE = 0.34, *t*(54) = 3.46, *p* = 0.001. The effects for both restrictions were reduced when the potential mediators experience of reactance and behavioral intentions had been added to the prediction, *b* = -0.70, SE = 0.29, *t*(54) = -2.39, *p* = 0.020 and *b* = -0.77, SE = 0.29, *t*(54) = -2.67, *p* = 0.010. The bootstrapped indirect effects of the illegitimate and the legitimate freedom threat via experience of reactance and behavioral intentions on anger were significant, *b* = 0.74, *SE* = 0.20, BC CI [0.32,1.44] and *b* = 0.71, *SE* = 0.22, BC CI [0.27,1.51], indicating that both restrictions first arouse an experience of reactance that further led to behavioral intentions to restore one’s freedom and finally resulted in a negative approach state, namely anger (for the path coefficients see **Figure 4**). Furthermore, the indirect effects of both threats on anger via experience of reactance were significant as well, *b* = 1.20, SE = 0.29, BC CI [0.55, 2.15] and *b* = 1.15, SE = 0.28, BC CI [0.54, 2.13], indicating experience of reactance and behavioral intention as mediators for both restrictions on anger.

These results support our hypothesis that behavioral intentions that aim to regain one’s freedom should result in approach motivation, positive or negative.

## Discussion

### Discussion of the Results

In the current research we explored effects of illegitimate and legitimate freedom threats in social interactions on people’s subsequent reactions to those threats. We hypothesized that although both illegitimate and legitimate freedom threats arouse the experience of reactance, they should differ in their physiological arousal effects. Supporting our hypotheses and confirming Brehm (1966) assumption, results indicated that both kinds of restrictions evoked experienced reactance and behavioral intentions to restore one’s freedom, for example by thinking about ruining the threatening man’s reputation or describing him as incompetent to others. Moreover, both restrictions led to the same amount of experienced anger, which is a key component of psychological reactance (Dillard and Shen, 2005).

However, with regard to people’s physiological arousal, our findings point to different underlying processes. While illegitimate freedom threats led to an immediate increase in HR, legitimate freedom threats led to a delayed increase in HR. We assume that illegitimate restrictions may be followed by a more automatic arousal. Legitimate restrictions, however, may first be followed by a more cognitive process and only after a delay be followed by a physiological arousal. Thus, people in the illegitimate condition may immediately feel their emotions boiling over. They are physiologically aroused and activated to “fight” for their freedom. People in the legitimate condition seem to need some more time to recognize the landlord’s unobvious restriction. After this delay in time they are able to enter the arousal state. *Dual process theories* might help to further understand the underlying mechanisms between these different processes. Dual process models distinguish between more impulsive, automatic and more reflective, controlled processes. As stated by Bargh (1994) automatic processes are typically unintentional and require little amounts of cognitive resources. Conversely, controlled processes are intentional and require considerable amounts of cognitive resources (see Gawronski and Creighton, 2013). Thinking of the landlord scenario, people in the legitimate condition may need more cognitive resources for understanding the restriction as a limitation of their freedom and for arguing against the restriction. Building on Strack and Deutsch’s (2004) reflective–impulsive model (*RIM*) of social behavior we thus propose that illegitimate restrictions are associated with the impulsive system, and legitimate restrictions are associated with the reflective system. However, both processes seem to be related to anger and counter arguing. In both restriction conditions participants show experience of reactance and behavioral intentions – as the intertwined model by Dillard and Shen (2005) suggests. If people are confronted with restrictions to their freedom, they feel the motivation to restore that freedom – no matter if the restriction has been illegitimate or legitimate. However, the process how to regain freedom might differ. Being confronted with an illegitimate restriction, participants immediately feel an aversive arousal. They feel their anger, experience reactance, and show behavioral intentions to restore their freedom. This may further lead to approach motivation and restore people’s agency. With regard to legitimate restrictions, however, the picture seems to be less clear. People do not like the restriction but there is an obvious and justified reason for the restriction. This could mean that people first have to reflect upon and argue against the restriction before getting into the same arousal state as people of the illegitimate restriction. When they are given the opportunity to restore their freedom, i.e., they intend to behave in a reactant way, positive approach motivation emerges. However, they experienced lower positive approach than people in the illegitimate condition. Yet, as this positive approach state has been achieved in both restrictions only after intending to behave in a freedom-restoring way, reactance seems to be functional in both restriction situations. These findings suggest that only after reactance has been aroused and demonstrated by behavioral intentions, one experiences a feeling of regained approach motivation. Deriving out of this, defensive behaviors may be important in order to experience one’s agency again (Jonas et al., 2014).

### Implications, Limitations, and Future Research

In the current study we were able to show that illegitimate and legitimate freedom threats share their outcomes – they both arouse similar amounts of experience as well as behavioral intentions. Moreover, they both evoke negative emotions (anger) and positive emotions with an approach motivational character. Most importantly, our findings highlight the usefulness of exploring people’s physiological reactions. By looking at people’s HR, we found important differences between the two threats. Those would not have shown by looking at behavioral indicators only. As Wright (2008) stated, physiological arousal is a good indicator of effort effects and as such predicts people’s motivation to achieve their goals (Brehm and Self, 1989). As we found a physiological difference between the two restrictions we also assume that people’s effort to restore their freedom differs. However, we did not test whether there are differences in people’s actual efforts but only measured intentional behavior. Future research might consider assessing real behavior in order to investigate people’s efforts to restore their freedom.

One further limitation of this study is that we did not include more of the relevant measures in a time sequence. For example, we did not measure negative (and positive) affect immediately after the freedom restriction but only following the reactance measures. Moreover, we did not incorporate a manipulation check for (il)legitimacy after both scenarios. For future research this would be useful to get a better insight into the timing of both cognitive and affective responses. Following the intertwined model research, counter arguing is one important part of the reactance process (Dillard and Shen, 2005; Rains, 2013). Therefore, the perception of (il)legitimacy could have changed during the reactance process. We suggested that participants in the legitimate restriction condition first had to think about the given situation embedded in the social interaction. The landlord did not want to let the flat to students because he had made bad experiences with students. However, participants could have tried to argue against the landlord’s reason not to hire the flat to students thinking of being a very proper and correct student himself/herself. Therefore it could be that those participants also had experienced a time-delayed arousal feeling of “illegitimacy” in the legitimate restriction^5^.

One might also ask whether the delayed physiological response to the legitimate restriction could be partly explained by the fact that more information had to be processed in the legitimate condition when thinking about the reasons provided for the landlord’s behavior. As this study is only a first step in understanding the underlying mechanisms and the considerations of the dual-process vs. intertwined model, future research should focus on shedding further light on the underlying mechanisms in illegitimate and legitimate restrictions. Strack and Deutsch (2004) show that both, the impulsive and reflective system, operate in parallel. However, the impulsive system enjoys priority over the reflective system because the latter only operates under conditions of sufficient cognitive capacity. The information processing in the impulsive system is assumed to be independent of resources. Consequently, we would predict that for example a cognitive load task (e.g., remembering a seven digit number) may influence legitimate (more “reflective”) but not illegitimate (“more impulsive”) restrictions of freedom.

When looking at the intertwined model of reactance (Dillard and Shen, 2005) in which reactance is defined as a combination of affect and cognition, one might be surprised that their definition misses a crucial factor: motivation. Brehm stated that reactance is “a motivational state and as such is assumed to have energizing and behavior-directing properties” (Brehm and Brehm, 1981, p. 98). Therefore, motivation is a very important factor that cannot be ignored. By adding the motivational components of physiological arousal and approach, our research attempts to better grasp the phenomenon of reactance.

During experiencing reactance and behaving in a reactant way, approach motivation seems to play an important role (see also Steindl et al., in preparation). It is defined as an “impulse to go toward” (Harmon-Jones et al., 2013, p. 291) and has been found in states sharing a motivational but not necessarily an emotional direction. Thus, anger (negative valence) and determination (positive valence) share the motivation to approach rather than avoid (e.g., Harmon-Jones et al., 2009, 2011). Believing in one’s ability to take action is a prerequisite for approach motivation to emerge (Harmon-Jones et al., 2003, 2006). As reactance motivates people to take action on restoring their freedom, they should feel capable to do so. Without the belief in one’s coping abilities, helplessness rather than reactance would emerge and thus, avoidance motivation would be more likely. As approach motivation is often assessed by frontal alpha asymmetry in EEG studies (e.g., Harmon-Jones et al., 2010), one could elaborate on the findings of this study and explore frontal alpha asymmetry in reactance processes. This would be especially interesting with regard to the question if illegitimate and legitimate freedom restrictions differ.

One critical limitation of our research is that we only used a scenario for the experimental manipulations to evoke reactance. One might question to what extent it is possible for participants to solely imagine how they might feel if they are restricted in their freedom. However, the findings of the current study reveal that in addition to indicating a strong experience of reactance and behavioral intentions to restore their freedom, participants even showed a physiological response – although they were confronted only with a scenario. Therefore, we assume that we were able to arouse reactance with scenarios only. However, it would be useful for future studies to replicate the present findings using real illegitimate vs. legitimate restrictions.

## Conclusion

In sum, the present study helped to get first interesting new insights into understanding the underlying mechanisms of illegitimate and legitimate restrictions of freedom. To better understand reactance processes, we added the important factors of motivation and physiological arousal (already mentioned by Brehm, 1966) to Dillard and Shen’s (2005) description of reactance as an intertwined affective and cognitive phenomenon. First, we showed that both illegitimate and legitimate freedom threats evoke similar amounts of reactance. Second, extending past research on reactance theory, we found that illegitimate restrictions lead to an immediate physiological arousal and legitimate restrictions lead to a time delayed physiological arousal. However, in order to provide a complete picture of the underlying processes of illegitimate and legitimate reactance processes and how these restrictions affect interaction processes, more research is needed. It is crucial to investigate the underlying processes of different forms of threat (illegitimate vs. legitimate) to enhance our understanding of social interaction processes.

## Footnotes

1 Items of the Experience of Reactance Scale: to what extent do you perceive the reaction of the landlord as a restriction of freedom? How much do you feel being put under pressure by his reaction? Are you frustrated about the reaction of the landlord? How inadequate do you think is his reaction? How much does his reaction bother you? How illegitimate do you think is the landlord’s reaction? To what extent are you offended by his reaction? Items of the Behavioral Intentions and Evaluation Scale: would you ever consider renting a flat of this landlord in the future? How much would you try to describe this man as incompetent to other students? How important would it be for you to argue against the landlord’s reaction? How strong is your wish to complain about his reaction at the professional association for tenant’s interests? How much would you advise other students against this landlord? Would you like to severely criticize the landlord in a daily newspaper? Would you like to ruin his reputation by publishing a negative review on a respective internet site? Do you think that this landlord could also have prejudices against foreigners? Do you think that the landlord also shows discriminatory behavior in other areas? How likely do you think it is that this man takes advantage of other people? We also measured the attractiveness of the apartment with one item and the sympathy for the landlord but did not use those two single items for further analyses. Participants in the legitimate (*M* = 1.94, SD = 0.64) and the illegitimate condition (*M* = 2.50, SD = 1.19) rated the apartment as less attractive compared to participants in the no restriction control group (*M* = 3.63, SD = 0.96), *F*(2,54) = 14.78, *p* < 0.001, $\eta_{\rho }^{2}$= 0.35. Furthermore participants showed less sympathy for the landlord in the two experimental conditions (illegitimate: *M* = 1.65, SD = 1.04; legitimate: *M* = 1.61, SD = 0.61) compared to the control group (*M* = 3.68, SD = 1.00), *F*(2,54) = 32.08, *p* < 0.001, $\eta_{\rho }^{2}$= 0.54.

2 For the measure of SC, we found neither a significant interaction (*F*(2,54) < 1, *p* = 0.703, $\eta_{\rho }^{2}$= 0.01) nor a significant main-effect of the restriction manipulation *F*(1,54) < 1, *p* = 0.420, $\eta_{\rho }^{2}$= 0.03; point of measure x restriction). Thus we did not include the measure in further analyses. Solely, we found a main-effect for the within factor (immediate vs. delayed response), *F*(1,54) = 31.98, *p* < 0.001, $\eta_{\rho }^{2}$= 0.37. Participants showed less SC at the second point of measure (delayed response, *M* = 0.35, SD = 0.53) compared to the first point of measure (immediate response, *M* = 0.80, SD = 0.90). However, this is not surprising, considering the fact that we used two sensors of NeXus 10, which only could be fixed with Velcro^®^strip on the fingers of the participants. This method is much more error-prone than the use of real electrodes, such as Beckman Ag/AgCI electrodes used by other biofeedback-instruments (Losch and Cacioppo, 1990; Eisenberg et al., 1991). However, this is not surprising considering the fact that we used two sensors of NeXus 10, which only could be fixed with Velcro^®^strip on the fingers of the participants. This method for measuring SC is much more error-prone than the use of real electrodes, such as Beckman Ag/AgCI electrodes used by other biofeedback-instruments (Losch and Cacioppo, 1990; Eisenberg et al., 1991).

3 The scale is based on Egloff et al. (2003), who originally proposed three positive factors: activation (active, attentive, inspired, alert), interest (interested, strong, determined), and joy (enthusiastic, excited, proud). However, due to a reliability of the interest factor of only α = 0.50 we combined the two factors activation and interest and termed it positive approach factor. Of the remaining three items of the positive PANAS scale we built the factor joy (enthusiastic, excited, proud; α = 0.84). Of the remaining seven items of the negative PANAS we built the factor negative emotions (distressed, scared, jittery, guilty, ashamed, nervous, anxious; α = 0.75). However, as approach motivation is a crucial concept in this article we only focus on the negative approach factor (anger) and the positive approach factor.

4 For explorative analyses we measured the subscales “Victim Sensitivity” and “Observer Sensitivity” from the “Justice Sensitivity Inventory” by Schmitt et al. (2005) as well as the “Collectivism and Individualism” scales by Triandis and Gelfand (1998).

5 Following Jonas et al. (2009) our measure of reactance also included one item measuring participants’ feeling of illegitimacy. When we ran a factor analysis for the seven experience of reactance items of our reactance measure we found that the “illegitimate”-item highly loaded on the extracted experience of reactance scale (0.73). Therefore, it is not surprising that following the time delay in which participants had the opportunity to counter-argue the freedom restriction they experienced about the same amount of “illegitimacy” in both restriction conditions.
